# Correlation of Malaria Rapid Test and Peripheral Blood Smear Microscopy among Patients attending Byumba Health Centre

**DOI:** 10.24248/eahrj.v6i2.700

**Published:** 2022-11-30

**Authors:** Cedrick Izere, Joyce Niyigena, Jean de Dieu Tuyishime, Alexis Nshimiyimana, Thierry Habyarimana, Pacifique Ndishimye, Callixte Yadufashije, Francois N. Niyonzima

**Affiliations:** a Department of Biomedical Laboratory Sciences, Ines-Ruhengeri Institute of Applied Sciences, Musanze district, Northern Province, Rwanda; b Department of Medical Research Center, Rwanda Biomedical Center, Kigali City, Rwanda; c Department of Public Health, Kampala University, Kampala, Uganda; d Department of Math, Science and PE, CE, University of Rwanda, Rwamagana-55, Rwanda

## Abstract

**Background:**

Malaria presents a diagnostic challenge in most tropical countries including Rwanda. Microscopy remains the gold standard for diagnosing malaria, however, it is labour intensive and depends upon the skill of the examiner. Malaria rapid diagnostic tests (MRDTs) have been developed as an easy, convenient alternative to microscopy.

**Methods:**

A cross sectional study was conducted from October to November 2019 on 130 febrile patients who were directed to the laboratory department for blood screening for malaria parasites at Byumba Health centre. The main objective of this study was to correlate Microscopy and MRDTs in diagnosis of malaria.

**Results:**

After signing a consent form, blood samples were collected and screened for malaria parasites microscopically and by using MRDTs. Data collection forms were filled with relevant information and obtained results for MRDTs and for peripheral blood smear were recorded. The collected data were statistically analyzed using GraphPad Prism 9 software. The mean age found to be 16 years old. In this study peripheral blood smear microscopy was considered as a reference method. The sensitivity and specificity of RDT Histidine–Rich Protein 2 (HRP-2) were calculated and found to be 96.6% and 60% respectively. The negative predictive value was found to be 92.85% where positive predictive value was 73.3%.

**Conclusion:**

MRDTs should be used along with microscopy to avert complications associated with delayed diagnosis and similar studies are required to identify alternative techniques with high specificity for the diagnosis of malaria.

## BACKGROUND

Malaria is one of the highest killer diseases affecting people in tropical countries especially in Africa. The 2019 World Health Organization (WHO) Malaria Report estimates that there were 228 million malaria cases and 405,000 deaths in 2018. According to WHO, the first priority for all countries where transmission rates of malaria are high or moderate is to ensure maximal reduction of morbidity and mortality through sustained provision of universal access to quality-assured and appropriate vector control measures, diagnostics and antimalarial medicines, while retaining the long-term vision of malaria eradication.^1^ Of all the human malaria parasites, *Plasmodium falciparum* (*P. falciparum*) is the most common pathogenic and is frequently fatal if untreated in time.^2^ Traditionally, in sub Saharan Africa, outpatients presumptively treat malaria based on patient's history of fever, however, different studies report that a significant proportion of patients treated this way may not be infected with malaria causing parasites (over 50% in many settings) and hence resulting into wastage of considerable amounts of drugs.^3^ However, this old clinical based practice is still relevant today especially, in cases involving infants where the time spent on getting a confirmatory laboratory diagnosis could lead to increased fatality.^4^ WHO currently makes the tentative recommendation that parasite-based diagnosis should be used in all cases of suspected malaria with the possible exception of children in high-prevalence areas and certain other situations.^5^ For this recommendation to be adhered to obviously, rapid and accurate laboratory finding or demonstration of malaria parasite should be established. The traditional method of microscopic identification of parasite however, is not only daunting in poor power setting, but also time consuming and requiring a lot of expertise/training. Thus, the peripheral blood smear examination technique is generally used, however, it is limited to large clinics/tertiary centres. This conventional staining of peripheral blood smears/microscopy still remains the gold standard in laboratory diagnosis of malaria.^4^ Malaria Rapid Diagnostic Tests (MRDTs) are commercially available in kit forms with all necessary reagents and the ease of performance of the procedures does not require extensive training or equipment to perform or to interpret the results. Results are read in 12 to 15 minutes. MRDT mainly come in two forms. One is antigen based and normally requires the use of haemolysed red blood cells while the other is antibody based and normally requires the use of extracted serum. Generally speaking, antibodies are better expressed in serum otherwise plasma could also stand in place of serum for antibody-based method.^6^ This study aimed to correlate Microscopy and MRDT (Histidine – rich protein 2 (HRP-2), Ag of *plasmodium falciparum*) in diagnosis of malaria at Byumba Health centre.

## METHODS

### Study Area

This study was conducted out in the laboratory department of Byumba Health Centre, located in Northern Province, Byumba Sector, Gicumbi District, Rwanda. The city lies about 60 kilometres (37 mi), north of the capital Kigali. This location lies approximately 30 kilometres (19 mi), south of the International border with Uganda at Gatuna.

### Study Design and Period

A cross sectional study design was conducted among patients of Byumba Health Centre. Data was collected from October to November 2019.

### Study population and Sample Size

130 Samples that tested for Malaria were used. The samples were taken from patients who attended Byumba Health Centre between October and November 2019.

### Inclusion and Exclusion Criteria

All male and female patients attending Byumba Health Centre between October and November 2019 with clinical suspicion of malaria based on fever and or history of fever within the previous 48 hours were eligible for inclusion in the study. Lack of consent and incomplete data constituted the exclusion criteria.

### Ethical Consideration

The study was approved by the research committee of Byumba Health Centre, accredited by Byumba District Hospital. The objectives and procedure were carefully controlled according to set Standard Operating Procedures (SOPs). Written consent was sought for from study participants or study participant's caretaker for minors. To ensure confidentiality, numbers were used as study participants' ID instead of names on patient's data extraction forms.

### Sample Collection and Processing

After filling the consent form, Blood sample was collected from the middle or ring finger of the patient by using lancet. 5μl of whole blood from the finger was added into MRDT, then 4 drops of assay diluents were added into MRDT according to the manufacture's protocol and test to detect malaria parasite/antibody detection method. The results were read after 15 min. Similarly, peripheral blood smears were made on a clean slide and allowed to air dry before being sent to the Parasitology laboratory. 10% Giemsa stain was used to stain thick smear for 15 minutes and tested with right microscopy. The results from both MRDT and microscopy were reported qualitatively (Positive or Negative). MRDT results and thick smear results were recorded on the data collection sheet. This was done within one hour from the collection time. Materials were consisted of Giemsa stain, microscopic slides, and light microscopy with good 100X objectives, MRDTs kits and lancets.

### Data Collection and Analysis

Collected data was checked and analysed using GraphPad Prism 9 software. The validity of diagnostic test was used in calculation and then the measurements were reported in number and percentage.

## RESULTS

### Demographic Characteristics of Study Subjects

The study considered a total of 130 participants; 70(54%) females and 60(46.1%) males. The mean age of the participants was found to be 16 years. The demographic characteristics of the study subjects are shown in Table 1;

**TABLE 1:** Demographic characteristics of the study participants

Characteristics	Gender		Total
Age (Years)	Female	Male	
1-24	47	28	75(57.6%)
25-34	10	18	28 (21.5%)
35-44	7	4	11 (8.4%)
45-54	4	6	10 (7.6%)
55-77	2	4	6 (4.6%)
**Total**	**70 (53.8%)**	**60 (46.2%)**	**130 (100%**)

**Proportions of malaria by MRDT and peripheral blood** smear microscopy are presented in table 2. True positive and true negative results were 44.6% and 40 % respectively while false positive and false negative results were 12.3% and 3.07% respectively.

**TABLE 2:** Malaria Status by MRDT and Peripheral Blood Smear's Microscopy

Positive for mRDTs & for microscopy	True positive=58 (44.6%)
Negative for mRDTs & for microscopy	True negative=52 (40%)
Positive for mRDTs & negative for microscopy	False positive=16 (12.3%)
Negative for mRDTs & Positive for microscopy	False negative=4 (3.07%)

### Sensitivity of MRDTs in Diagnosis of Malaria

In this study, microscopy was considered as a method of reference. The sensitivity of MRDTs HRP2 in diagnosis of malaria is reported at 96.6%, this is presented in Table 3. Therefore, 44.6% of the patients who tested positive with both methods (peripheral bold smear microscopy and MRDTs) were considered true positives. Patients who tested negative with MRDT and positive to peripheral blood smear microscopy were 3.07% and these were considered false negative. This high sensitivity (96.6%) may be due to factors relating to how health community workers transported the MRDTs, thus being damaged by extreme temperature or humidity during transportation, and storage. These results are in contradiction with what was reported by a study elsewhere, where the sensitivity was 76.9%.^15^

**TABLE 3:** Sensitivity of MRDTs (HRP-2) in Diagnosis of Malaria

Variables & Formula	Values
True positive	44.60%
False negative	3.07%
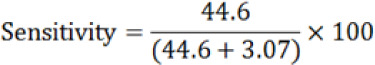	96.60%

### Specificity of MRDTs in Diagnosis of Malaria

Table 4 shows the specificity of MRDT (HRP-2) at 76.4% in diagnosis of malaria. True negative was 20.45% (patients who tested negative by both MRDT and peripheral blood smear microscopy), whereas the false positive was 12.3%, (patients who tested positive with MRDTs but tested negative with peripheral blood smear microscopy). Transportation of MRDTs, sample correction, storage and humidity could be the factors responsible for the low specificity and this result is in contradiction with what was reported in a similar study elsewhere where the specificity was 94.2%.^16^

**TABLE 4:** Specificity of MRDTs (HRP-2) in Diagnosis of Malaria

Variables & Formula	Values
True Negative	40.0%
False Positive	12.3%
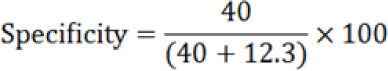	76.4%

### Predictive Values of MRDTs in Diagnosis of Malaria

Predictive values of MRDT (HRP-2), positive and negative were calculated as shown in Table 5. Negative predictive value was found to be 92.87% whereas positive predictive value was 78.38%. The negative predictive values of MRDTs were high compared to the positive predictive values and were in contradiction to the study conducted in Egypt which was 96.2%.^13^ These results mean that if you tested negative for Malaria by MRDT (HRP-2), you would have 92.85% chances of not having the disease. When you tested positive for Malaria with MRDT (HRP-2), you would have a chance of 73.3% of truly having the disease.

**TABLE 5:** Positive and Negative Predictive Values of MRDT in Diagnosis of Malaria

Variables & Formula	Values
True negative	40.00%
False negative	3.07%
True positive	44.60%
False positive	12.30%
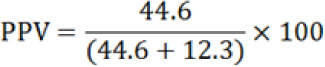	78.38%
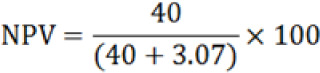	92.87%

PPV: Positive predictive values   NPV: Negative predictive values

## DISCUSSION

Malaria affects a significant number of people across the world each year and is the most wide-spread parasitic disease encountered.^7^ The disease has a worldwide distribution and is found throughout the tropics, sub-Saharan Africa, South East Asia, the Pacific islands, India, Central and South America. Malaria caused by *Plasmodium falciparum* predominates in Africa where the mortality attributed to it approaches 1 million annually, and accounts for 90% of the global malaria burden.^8^ Majority of these deaths are of children under the age of 5 years. Thus, one child dies of malaria in Africa every 30 seconds, which translates into a tragic 3000 children each day. Many of the children who survive an episode of severe malaria suffer from brain damage and cognitive disability, consequently crippling these families with its debilitating aftermath.^9^ Malaria presents a diagnostic challenge in most tropical countries like Rwanda. Microscopy remains the gold standard for diagnosing malaria, but it is labour intensive and depends upon the skill of the examiner. RDTs have been developed as an easy, convenient alternative to microscopy.^10^ Poor diagnosis of malaria implies under diagnosis and inappropriate treatment procedures.^3^ MRDTs are known to capture at least 3 target antigens: lactate dehydrogenase (LDH), *Plasmodium falciparum* histidine-rich protein 2 (PfHRP2) and pan-plasmodial aldolase. HRP-2 MRDTs are the most sensitive for parasite detection and are heat-stable under field conditions compared to the other antigen tests.^12^ However, HRP-2 MRDTs have limitations, as their performance has been shown to be affected by product quality and parasite-related factors such as *pfhrp2/3* gene deletion, non-*P*.*falciparum* species and prozone effects that may lead to false-negative MRDTs.^13^ Microscopy is the most widely tool used to diagnose malaria at peripheral levels. In capable hands it is very sensitive for parasitaemia ≤50/μL (0.001%)^19^ and it can give important information to the clinician like species, parasites stages and parasite density.

The observed high sensitivity (96.6%) and specificity in this study is similar to reports of other studies conducted elsewhere.^17, 18^ The study also observed high negative predictive values of MRDTs compared to the positive predictive values. This finding is in contradiction with findings of a similar study conducted in Egypt.^13^ Several studies conducted elsewhere have shown varrying degree of false negative result for MRDT because of hyperparasitaemia, deletion or mutation of *HRP-2* gene and the prozone effect (which is defined as false-negative or falsely low results in immunological reactions because of excess of either antigens or antibodies).^20,21^

## CONCLUSION AND RECOMMENDATIONS

This research on the correlation of malaria rapid test and peripheral blood smear microscopy has been carried out on patients who attended Byumba Health Centre, suspected to have malaria. The sensitivity of HRP-2 based Rapid diagnostic test for malaria was (96.6%) and high to the specificity of this type of MRDT with 76.4%. The negative predictive values of MRDTs were high compared to the positive predictive values. If you tested negative for Malaria by MRDT (HRP-2), you would have 92.87% chances of not having the disease. When you tested positive for Malaria with MRDT (HRP-2), you would have a chance of 78.38% of truly having the disease. In this study, there aretests that were considered as false positive (positive for MRDTs and negative for peripheral blood smear microscopy) while Other tests were reported as false negative (negative for MRDTs and positive for peripheral blood smear microscopy). The results obtained by MRDT (HRP-2), for malaria parasites should be confirmed with other Tests of high specificity such as microscopy and Polymerase Chain Reaction. Health professionals are recommended to confirm MRDTs results with microscopy before administering treatment and precautions on the uses of MRDTs, transportation of kits and samples correction should be taken into consideration. Further studies should determine the most appropriate type of malaria diagnostic test to be used in combination with microscopy and MRDTs.
